# Identification of essential proteins based on a new combination of topological and biological features in weighted protein–protein interaction networks

**DOI:** 10.1049/iet-syb.2018.5024

**Published:** 2018-12-01

**Authors:** Abdolkarim Elahi, Seyed Morteza Babamir

**Affiliations:** ^1^ Department of Software Engineering University of Kashan Kashan Iran

**Keywords:** proteins, drugs, biology computing, ontologies (artificial intelligence), topology, biological features, weighted protein–protein interaction networks, network topology‐based centrality measures, low‐connectivity essential proteins, topological centrality measures, protein complexes, incomplete PPI networks, weighted centrality measures, YHQ‐W dataset, YMBD‐W dataset, YDIP‐W dataset, YMIPS‐W dataset

## Abstract

The identification of essential proteins in protein–protein interaction (PPI) networks is not only important in understanding the process of cellular life but also useful in diagnosis and drug design. The network topology‐based centrality measures are sensitive to noise of network. Moreover, these measures cannot detect low‐connectivity essential proteins. The authors have proposed a new method using a combination of topological centrality measures and biological features based on statistical analyses of essential proteins and protein complexes. With incomplete PPI networks, they face the challenge of false‐positive interactions. To remove these interactions, the PPI networks are weighted by gene ontology. Furthermore, they use a combination of classifiers, including the newly proposed measures and traditional weighted centrality measures, to improve the precision of identification. This combination is evaluated using the logistic regression model in terms of significance levels. The proposed method has been implemented and compared to both previous and more recent efficient computational methods using six statistical standards. The results show that the proposed method is more precise in identifying essential proteins than the previous methods. This level of precision was obtained through the use of four different data sets: YHQ‐W, YMBD‐W, YDIP‐W and YMIPS‐W.

## 1 Introduction

Essential proteins are vital for the life and/or reproduction of organisms, and play a very important role in maintaining cellular life. If the destruction of a certain protein would cause lethality or infertility, it can be said that it is essential to an organism, that is, the organism could not survive without it [1]. Compared with non‐essential proteins, essential proteins are more likely to remain in biological evolution [2]. For example, essential proteins are excellent targets for the development of new potential drugs and vaccines for the treatment and prevention of diseases. There are methods that can be used to determine essential proteins. First, there are experimental methods such as RNA interference [3], single gene knockouts [4], and conditional knockouts [5]; these require considerable amounts of time and resources and are not always applicable. The second set of methods consists of computational methods, which are faster and cheaper than experimental approaches. The computational methods that have so far been used to determine essential proteins can be divided into the following basic categories:

- Single centrality methods include degree centrality (DC) [6], betweenness centrality (BC) [7], subgraph centrality (SC) [8], eigenvector centrality (EC) [9], network centrality (NC) [10] and local average connectivity (LAC) [11], which can be used due to abundant access to protein–protein interaction (PPI) network data. High degree (hub) proteins in the network are more likely to be essential [6]. Since the degree of a vertex or a hub is noted in PPIs, most authors have confirmed the relationship between the DC and essentiality of proteins [12], and some have examined the reasons behind link [13]. However, according to the centrality–lethality rule, proteins with more network associations are more likely to be essential. However, there are significant amounts of low‐connected proteins in the network that are essential. This method also performs poorly in incomplete PPI networks. Since PPI networks are produced using high‐throughput methods, they have a large number of false‐positive interactions; this feature also somewhat decreases the level of precision when predicting essential proteins. Some protein interactions are more important than others. Moreover, false‐positive interactions behave differently from true interactions.
  Second, there are methods that use a combination of centralities such as LBCC [14] (local density, BC and in‐DC of the complex), which is considered as one of the most effective recent methods in this category. Chau *et al.* suggested a combination of centrality measures for the identification of essential proteins [15]. Del *et al.* analysed 18 reconstructed metabolic networks using 16 different centrality measures, and found that any paired combination of single centrality measures can improve the efficiency of predictions; however, three or more combinations had no effect on improving the precision of predictions [16]. Qin *et al.* [14] proposed a technique called LBCC to explore essential proteins based on the topological properties of the network using four datasets: YMIPS, YHQ, YDIP and YMBD. The experimental results implied that the efficiency of LBCC is superior to methods that only use a single centrality, such as DC, EC, LAC, SC, BC and NC. It is also better than the LIDC method [17] (local interaction density combined with protein complexes), which uses a combination of several centralities. This method focuses on the size of the topology centrality, and has little predictive power for low‐connectivity essential proteins. However, in our proposed method, we compensated for this shortcoming by including biological features to detect low‐connectivity essential proteins. We compared our proposed method with the LBCC method; the results show that our proposed method leads to an acceptable increase in the precision of predicting these essential proteins.
- Methods that measure biological characteristics include Go_ELAC [18] (local network topology and gene ontology (GO) and PMN (based on the integration of weighted interaction networks and functional modules) [19]. Hart *et al.* showed that, according to their experimental data, a large number of essential proteins tend to accumulate in specific protein complexes [20]. Proteins can interact with each other where is detected in biological experiments using co‐expression and based on gene expression data in the duration of time. Zhao *et al.* proposed a method called PEMC to combine the topology of the network with a gene profile and domain information for construction of weighted protein networks in order to discover essential proteins [21]. Li *et al.* presented a new method for the construction of protein interaction network (PIN) using gene expression profiles and sub‐cellular location information. They found that if two proteins are not expressed at the same time within the cell or not placed in a similar compartment of the cell, their interaction could not occur. From the spatial point of view, the dynamics of protein expression indicates that the protein performs its function in some cellular compartments [22]. Li *et al.* proposed a new method called SPP based on sub‐network partition and prioritisation by integrating sub‐cellular localisation information. They found that their method can significantly reduce the effect of false positives and increase the prediction accuracy (ACC) of the essential protein compared to existing computational methods such as DC, BC, SC, EC, IC and NC [23]. Shang *et al.* employed RNA‐Seq data instead of microarray gene expression profiles to increase the ACC of prediction [24]. Lei *et al.* presented a new method called RGS based on RNA‐Seq, subcellular localisation information and Go annotation data. They measured the network by the Go terms information and Pearson correlation coefficient of RNA‐Seq [25]. Li *et al.* proposed a new method called GOS for the combination of the expression data, ontology and sub‐cellular localisation information in order to identify the essential proteins [26]. Li *et al.* proposed a method whereby calculating the topological potential value of each protein, one can provide more accurately the importance of each protein from the PPI network [27]. Peng *et al.* combined localisation specificity (SP) for essential protein detection with eight centrality methods separately to calculate localisation SP centrality scores for proteins based on the protein sub‐cellular localisation interaction network. Compared with the high‐ranking centrality method, more proteins with high LCSs from PSLINs were detected as essential. They proposed that proteins should be localised in their sub‐cellular compartments until their expected functions could be performed, so that sub‐cellular localisation information could be useful for identifying essential proteins [28]. Liu and Luo [18] presented an algorithm called GO_ELAC, with the aim of identifying essential proteins by integrating network topology and GO. The experimental results show that the efficiency of the GO_ELAC approach is better than methods that only use a single centrality, such as DC, CC and LAC. It is also superior to maximum neighbourhood component and density of maximum neighbourhood component, which considers, respectively, the size and maximum density of the connective component as the vertex rank. Our proposed method, in addition to using centrality measures and GO, examines the neighbourhood on more levels, as well as biological properties, compared to the GO_ELAC method. Yi *et al.* [19] also developed a technique called PMN to identify essential proteins based on functional modules and weighted PPI networks. Using this technique, they examined the tendency of essential proteins to fit into functional modules as a biological feature of the proteins. Luo and Qi [17] introduced a method called LIDC to detect essential proteins based on local interaction density and protein complexes. The experimental results obtained from the YMIPS dataset show that the performance of LIDC is superior to the nine methods: DC, BC, NC, LID [17], PeC [29], CoEWC [30], WDC [31], ION [32] and UC [33]. We compared our proposed method with the LBCC method, and this method compares its results with the LIDC method. The experimental results show a significant improvement in prediction precision for LBCC and our proposed method compared with LIDC and LBCC, respectively. Li *et al.* [29] presented a method called PeC by combining the edge clustering coefficient with the correlation coefficient of gene expression data. The experimental results showed that the PeC method worked very well for the prediction of essential proteins compared to the previous 15 proposed methods in the *Saccharomyces cerevisiae* network. This method has been compared with the LIDC method. According to [17] and the above analysis, our proposed method also significantly improves the precision of predictions compared to the LBCC method, and consequently, the LIDC and PeC methods. Although these methods are considered to have led to improvements in prediction, it is still possible to significantly increase the precision of predictions. This is our motivation for this study.

In this study, we use a combination of centralities, as well as measuring the biological characteristics of essential proteins. The input network was weighted in the proposed method in order to eliminate false‐positive interactions. By measuring the semantic similarity between pairs of proteins based on GO, the edges of low certainty were deleted before the creation of a weighted network. To improve the size of the local centrality in the next step, the precision of prediction was assisted using the idea of neighbourhood development at the second and third levels of indirect neighbourhoods, as well as their levels of significance. Next, the level of precision was significantly raised by including the biological characteristics of essential proteins, such as the tendency of essential proteins to link with others, the necessity of their presence in protein complexes and the assessment of their significance in the final classifier. All of the combinations were examined using binary logistic regression in order to calculate the significance level. Due to the above‐mentioned factors, this study has recommended a new method to identify essential proteins based on a combination of the best representative topological measures of local and global centrality on the one hand, and measures of the biological characteristics of essential proteins in weighted PPI networks on the other. Several experiments were conducted on modified PPI networks in *Saccharomyces cerevisiae*, YHQ‐W, YMBD‐W, YMIPS‐W and YDIP‐W, as described in Section 4.1. The proposed method was compared with three previous ones that only used a single centrality, the most efficient recent combinational centrality methods in the second category (such as LBCC) and the most recent methods in the third category, such as Go_ELAC, which uses the biological characteristics of essential proteins [18] and PMN [19].

The experimental results show that a combination of local and global centrality criteria measures, network topology and measures of the biological characteristics of essential proteins increases the precision of prediction by >10%.

## 2 PPI network modelling

The networks used in this research are non‐directional and weighted. With the graph $G=\left(V,E,W\right)$, it has been shown that $V=\left\{V1,V2,\ldots ,Vn\right\}$ is a series of vertexes and $E=\left\{e1,e2,\ldots ,en\right\}$ is a set of edges with *W* as the weight of each edge. Each edge $\left(Vi,Vj\right)$, weighted as $W_{i,j}$, shows the strength of the interaction between the vertices $V_{i}$ and $V_{j}$ based on semantic similarity as a result of GO. The computations for the PPI networks were made using the GFD‐Net app [34] (analyse a gene network based on GO and calculate a quantitative measure of its functional dissimilarity), a plugin of Cytoscape tool in the weighting of some datasets and followed by deleting false‐positive interactions in these networks. The subtraction of ‘functional dissimilarity’ from 1 led to the calculation of the ‘functional similarity’ value, called the interaction strength, and the weight between proteins pairs. The retrieved information can be observed from GO after the analysis of dissimilarity.

In the field of graph theory and network analysis, there are a number of measures to help determine the importance of vertices. This section defines the measures of some well‐known centralities.

The weighted DC includes vertex *i* and the total weight of the edges connecting vertex *i* to its neighbours (1) [35] where $N_{i}$ is a set of all the neighbours of vertex *i*

(1) $$\text{DCW}\left(i\right)=\sum_{j\in N_{i}}W_{i,j}$$

In the weighted BC BCW(*i*), vertex *i* is the average value of the shortest path through vertex *i* (2) [35]

(2) $$\text{BCW}\left(i\right)=\sum_{s}\sum_{t}\frac{\sigma_{st}\left(i\right)}{\sigma_{st}},\;s\neq t\neq i$$

so that $s_{st}$ is the total number of shortest paths between vertices *s* and *t*; $\sigma_{st}\left(i\right)$ refers to the number of shortest paths from vertex *s* to vertex *t* passing through vertex *i*.

In the weighted closeness centrality CCW(*i*) of a vertex *i* can be defined in a weighted graph *G* as (3) [35]

(3) $$\text{CCW}\left(i\right)=\frac{1}{\sum_{j\neq i}C_{p}\left(i,j\right)}$$

where $C_{p}(i,j)$ is the cost of the shortest path *p* from node *i* to node *j* and CCW(*i*) is a global measure that describes the way that vertex *i* connects to other vertices. To calculate the cost of the shortest path $C_{p}(i,j)$, first, the cost of the edge between vertex *i* and vertex *j* is considered $C_{i,j}=1/W_{i,j}$ and then the sum of the cost of all of the edges are obtained. If there are multiple paths from vertex *i* to vertex *j*, the shortest path is the one with the lowest cost; the shortest path cost is shown as $C_{p}(i,j)$.

The weighted EC ECW(*i*) of vertex *i* in the weighted graph *G* is defined as the *i* th component of the principal eigenvector of ***A***, where ***A*** is an edge weight matrix. If ρ is an eigenvalue and ***e*** is an eigenvector, for equation re=Ae, we can obtain $\text{ECW}\left(i\right)=e_{1}\left(i\right)$, which $e_{1}$ corresponds to the largest eigenvalue of ***A*** [35].

In weighted network *G*, the LAC‐based centrality LACW(*u*) of vertex *u* is defined as (4) [36]

(4) $$\text{LACW}\left(u\right)=\frac{\sum_{s\in N_{u}}\sum_{t\in N_{s}\cap N_{u}}w\left(s,t\right)}{\left|N_{u}\right|}$$

for given node *u*, $N_{u}$ denotes the set of its neighbours and $\left|N_{u}\right|$ does the number of its neighbours. Parameter *w* (*s*, *t*) is the weight of the edge connecting node *s* and node *t*. Parameter *s* is one of the neighbours of node *u*, and *t* is the neighbour of *s* in $N_{u}$.

## 3 Proposed method

This section presents a method called DENCI, which applies a combination of weighted degree measures of the vertex and its next neighbours, a weighted measure of each protein in a complex set, and the measure of its association with important proteins, according to the following concepts:

- Since we know that essential proteins tend to form in very connected clusters, neighbourhood has been used on more levels. That is the reason why we used the new independent variable DWNN to get more neighbourhoods.
- Since the essential proteins gather in protein complexes, we used the MCLUS independent variable to measure the importance of proteins in protein complexes.
- Both local and global properties are important in identifying essential proteins, so we used two independent variables, LACW and DWNN.
- There are many low‐connectivity proteins that are also essential and that are ignored by methods based on topological properties. For example, in the DIP‐core dataset, there are 685 essential proteins. While 438 essential proteins are presented as essential low‐connectivity proteins with degree ≤5 (∼63.9%), 247 essential proteins are high‐connectivity essential proteins (∼36.1%) [37]. So, we tried to detect low‐connectivity essential proteins using their biological properties, which increased the precision of identification (thanks to the new MCLUS and Importance independent variables).

Two computational methods are usually performed to discover the essential proteins, one which uses network topology and the biological characteristics of essential proteins. However, many low‐connectivity proteins are also known to be essential proteins. Thus, the identification of low‐connectivity essential proteins is performed using biological methods. We inserted all of the individual measures (independent variables) in the logistic regression analysis into the SPSS software; those with more predictive power were considered in the construction of the final equation. We removed any that were not significant (P−value>0.05).

The weighted edges indicate the strength of the interaction between two proteins in the network graph. Therefore, by using weighted measures of centrality, a greater number of essential proteins can be detected than with centrality measures in non‐weighted networks. Here, first, the network is weighted by the Cytoscape app GFD‐Net to measure the semantic similarity between pairs of proteins based on GO (a biological process). Then, the topological properties of the graph are used to determine the importance of each vertex. The vertex degree is considered one of the most well‐known measures for detecting essential proteins. Degree centrality is usually used for local measures, but this criterion is a poor indicator of connections in the network and most of the literature has noted that centrality measures alone cannot correctly predict all essential proteins. So, we use a combination of rules to increase the precision of essential protein diagnosis.

**Fig. 1:**
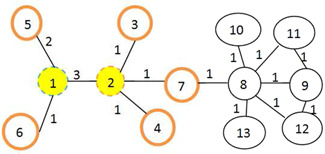
Vertexes 1 and 2 having an equal DC but with various topological properties

According to Fig. 1, vertices 1 and 2 are equal in weighted degrees but are topologically different both in terms of local properties (number of interactions in 1 compared to 2) and global characteristics (different positions of 1 relative to 2 throughout the entire network).

In a measure such as this, the importance of each vertex is assessed through the combined total weighted of each vertex (weighted degree (DCW)) as the local property, and the weighted BC (BCW) is appraised as the global characteristic

(5) $$\text{CWD}\left(x\right)=\text{DCW}(x)\times \text{BCW}(x)$$

However, even when two vertices have the same number and strength of interactions but are topologically different, then the number and strength of interactions cannot be compared properly when identifying essential proteins (Fig. 1). Therefore, more neighbourhood levels were examined to detect the topological differences in vertices. On the other hand, proteins tend to be densely associated with neighbouring vertices in order to implement a particular biological function, and essential proteins fall into these dense modules. The fundamental vertices in the modules maintain the existing modules and their biological functions. Therefore, the forms of modules can also be incorporated into the identification of essential proteins through the use of neighbourhoods. Since essential proteins tend to fit into strongly associated clusters [13], the neighbourhood can be raised at more levels (Fig. 1). Equations (6) and (7) define the use of neighbourhood measures and local and global topological characteristics in the diagnosis of essential proteins, Therefore, adjacency(*x*) shows the set of nearest and next nearest neighbours of vertex *x*, and DW(*x*) is the sum of ‘weighted DC multiplied by the BC (as in (5)) of vertex *x*’ and nearest and next nearest neighbours

(6) $$\text{DW}\left(x\right)=\sum_{y\in \text{adjacency}\left(x\right)}\text{CWD}\left(y\right)$$

(7) $$\text{DWNN}\left(y\right)=\sum_{x\in \text{adjacency}\left(y\right)}W_{y,x}\times \text{DW}\left(x\right)$$

Tests by the software IBM SPSS Statistics using binary logistic regression on the neighbourhood measure revealed that indirect neighbourhood at two subsequent levels is significantly better than at one subsequent level. The coefficient $W_{y,x}$ shows the power of the interaction between proteins and it is calculated on the edge of the network as the edge weight. We obtained this value by implementing the Cytoscape app GFD‐Net. Using this app, the level of dissimilarity of the two interacting proteins was calculated based on GO data. We then subtracted it from 1 to obtain the similarity value (see Fig. 1).

Vertex 1 has three neighbours, namely 2, 5 and 6, so the adjacency is equal to $\text{adjacency}\left(1\right)=\left\{2,5,6\right\}$ and $\text{DWNN}\left(1\right)=2\times \text{DW}\left(5\right)+1\times \text{DW}\left(6\right)+3\times \text{DW}\left(2\right)$. To calculate each of the DWs according to (6)

$$\begin{array}{rl} \text{DW}\left(5\right) & =\text{CWD}\left(6\right)+\text{CWD}\left(2\right)+\text{CWD}\left(5\right)\\ & \;+\text{CWD}\left(3\right)+\text{CWD}\left(4\right)+\text{CWD}(7)=\text{BCW}\left(6\right)\times 1\\ & \;+\text{BCW}\left(2\right)\times 6+\text{BCW}\left(5\right)\times 2+\text{BCW}\left(3\right)\times 1\\ & \;+\text{BCW}\left(4\right)\times 1+\text{BCW}(7)\times 2 \end{array}$$

where for instance, BCW(5) shows the weighted BC of vertex 5, and factor 2 is the weighted DC of this vertex. As such, the values of DW(6) and DW(2) are obtained and included in (7). Vertex 5 has only one neighbour called vertex 1, in which direct and indirect neighbours of vertex 1 are vertexes 6, 2, 5, 3, 4 and 7.

In PPI networks, there are generally functional modules that play a key role in biological functions and essential proteins, compared to individual proteins, tend to be within these functional modules [20]. At this stage, the ranks of all proteins are calculated, taking into account measures of the essential biological characteristics of essential proteins and clusters. We obtained clusters by implementing the Cytoscape app MCODE. The rank of protein *x*, *R* (*x*), is defined as the total weighted degree of vertex *x* with all of the proteins in clusters containing protein *x*. If vertex *x* does not exist in any clusters, then vertex *x* is considered as a non‐essential protein with an *R* (*x*) value of zero. If $CP=\{cp_{1},cp_{2},...,cp_{n}\}$, and a set of protein complexes contain protein *x*, then the weighted sum of this protein compared to the other proteins within those complexes is defined as the measure of MCLUS(*x*) protein

(8) $$\begin{array}{rl} \text{MCLUS}(x)= & \sum W_{x,y}|x,y\in \left\{{cp}_{1},{cp}_{2},...,{cp}_{n}\right\}\\ & \text{and}\;y\in \text{adjacency}\left(x\right) \end{array}$$

In this section, we propose types of biological features that can be used to detect essential proteins. One biological feature of essential proteins is known to be the relationship between essential proteins and other important proteins, meaning that if a protein has a stronger relationship with other proteins, one can conclude that the protein has a significant preference and is, therefore, a better candidate for being an essential protein. The procedure for selecting important proteins (SIP) is described below:

1. Initialise the dataset of the important protein, SIP = {Null}.
2. Calculate the weighted degree of each protein in the dataset.
3. Find the protein with the largest weighted degree (P_max) in the dataset and add it to the SIP.
4. Remove P_max and its neighbouring proteins from the dataset.
5. Return to step 3 and repeat until the dataset is empty.
6. Output the SIP.

Afterwards, we calculate the weighted degree between each protein and its neighbours, provided that the SIP has neighbours. Therefore, we compute the importance of each protein in terms of its relationship with other significant proteins and we show this as Importance(*x*)

(9) $$\begin{array}{rl} \text{Importance}\left(x\right) & =\sum W_{x,y}|y\in \text{adjacency}\left(x\right)\\ & \text{and}\;y\in \left\{\text{SIP}\right\} \end{array}$$

Binary logistic regression was used to examine all combinations of the various centrality measures using three measures: neighbourhoods at the second and third levels (DWNN), MCLUS and importance. In two datasets, namely YMBD and YHQ, the weighted local average centrality (LACW) was highly significant in comparison with the centrality measures of DCW, BCW, ECW, CCW, NCW, SCW and ICW. Finally, these four combinations detected higher numbers of essential proteins.

The essentiality of proteins can be judged using ratings. Before ratings were given, all of the measures were normalised using maximum–minimum normalisations, according to which all values between 0.0 and 1.0 were linearly transformed. This is a simple way of comparing levels on different scales and using different measurement units. If $\max(x_{ij})$ and $\min(x_{ij})$, are the maximum and minimum of the *j* th feature, respectively, and $X_{ij}$ is the measure of protein *i* at the centrality and feature of *j*, then the normal values of the measures are obtained as

(10) $$\text{NM}\left(X_{ij}\right)=\frac{X_{ij}-\min\left(X_{ij}\right)}{\max\left(X_{ij}\right)-\min\left(X_{ij}\right)}$$

Therefore, the proposed method is defined by combining the above concepts as

(11) $$\begin{array}{rl} \text{DENCI}\left(x\right) & =a\times \text{DWNN}\left(x\right)+b\times \text{MCLUS}\left(x\right)\\ & \;+c\times \text{Importance}\left(x\right)+d\times \text{LACW}\left(x\right) \end{array}$$

where *a*, *b*, *c* and *d* show the coefficients for the Importance of each calculation, which are part of essential protein identification; the functions DWNN, MCLUS and importance have also been described above. The information to the logistic regression is fed through 19 independent variables that use three columns of the dataset. Two columns are related to the name of the proteins and a column is related to the weight of the interactions between them. Values of 16 independent variables (weighted and unweighted centrality measures) are obtained using the Cytoscpe software along with the CytoNCA plugin, whose input information is the dataset that has the same three columns. Three other independent variables were also implemented by the C# language, which their values were obtained through the network, or the three data columns, and some of the centrality measures obtained from the CytoNCA. For fitting the binary logistic regression model, first, the response variable was converted to a binary response variable. Then, values of 19 independent measure variables were inputted to the model (16 variables for weighted and unweighted centrality measures, two variables for biological properties and one for the combination of local and global centrality measures for neighbours of higher levels). The results of the Wald test and its comparison with the significance level of 0.05, indicated that only the variables DWNN, MCLUS, importance and LACW (four independent variables) were significantly related to the type of protein (whether it was essential or non‐essential). Since the value of $R^{2}$ for the four above‐mentioned combinations was >5, the best combination of independent variables was finally selected. The value of parameters *a*, *b*, *c* and *d* was obtained using value $B_{i}$, which can be verified according to the significance level (Sig) and the Wald test. The influence of each of the parameters *a*, *b*, *c* and *d* was determined using its Wald value. We normalised value of four independent variables based on (10), before conducting experiments using binary logistic regression in order to improve value of the four parameters. For example, the parameter values and its Wald values were: [(*a*  = *B*_1_  = 0.221, Wald = 27.902, Sig = 0.000), (*b*  = *B*_2_  = 0.338, Wald = 40.531, Sig = 0.000), (*c*  = *B*_3_  = 0.270, Wald = 31.120, Sig = 0.000) and (*d*  = *B*_4_  = 0.189, Wald = 25.203, Sig = 0.000)] for the YMBD‐W dataset. For the YMIPS dataset, the parameter values and its Wald values were: [(*a*  = *B*_1_  = 0.239, Wald = 30.403, Sig = 0.000), (*b*  = *B*_2_  = 0.314, Wald = 39.945, Sig = 0.000), (*c*  = *B*_3_  = 0.298, Wald = 35.172, Sig = 0.000) and (*d*  = *B*_4_  = 0.148, Wald = 12.837, Sig = 0.009)]. Due to the large number of records (samples) in our input datasets, if the samples are increased, the proportions of the coefficient values do not change and remain stable. With the increase of the numbers of samples, very low changes were observed in the coefficients; by considering a large number of samples in our input datasets, the changes were low and the presence of such changes is normal.

## 4 Results and discussion

### 4.1 Experimental data

In this study, *S. cerevisiae* was used as the experimental organism to evaluate the efficiency of the proposed method, DENCI, because this organism has the most complete data. The PPI network datasets were collected from the MIPS database [38], the DIP dataset [39], and two datasets from the website of Gerstein Lab (Gersteinlab.org). Accordingly, four datasets were obtained from the MIPS database, Gerstein Lab and the DIP dataset, before being weighted and named as YMIPS‐W, YMBD‐W, YHQ‐W and YDIP‐W, respectively, after the removal of false‐positive interactions. The dataset YMIPS‐W consists of 4199 proteins with 11,656 interactions. YMBD‐W was selected from the original datasets of MIPS, BIND and DIP, and comprises 2242 proteins and 10,260 interactions. YHQ was compiled by Yu *et al.* [40], and was named YHQ‐W after being weighted. It contains 4291 proteins with 21,150 interactions. The dataset YDIP‐W also includes 4786 proteins with 24,216 interactions. The essential proteins of *S. cerevisiae* were collected from the MIPS [38], Saccharomyces Genome Database (SGD) [41], Database of Essential Genes (DEG) [42] and Saccharomyces Genome Deletion Project databases [1]. A protein is known as an essential protein if it exists at least in one essential database. The details of the datasets are presented in Table 1.

**Table 1:** Information on the four PPI datasets: YHQ‐W, YMBD‐W, YMIPS‐W and YDIP‐W

Dataset	Proteins	Interaction	Essential proteins
YMIPS−W	4199	11,656	973
YMBD−W	2242	10,260	712
YHQ−W	4291	21,150	1056
YDIP−W	4786	24,216	1122

The protein complexes were directly obtained from four protein complex datasets, namely CM270 [38], CM425 [43], CYC408 and CYC428 [44, 45], which contained 745 protein complexes (in comparison with 2167 proteins). These four real protein complexes were collected within an integrated protein complex. Only those complexes with over two vertices are retained in a complex protein. CM270 is a gold standard protein complex downloaded from the MIPS database [38], which includes 270 complexes and 1230 proteins. CM425 [43] was integrated from the MIPS database [38], Aloy *et al.* [46] and the SGD database [47] contained 425 complexes and 1970 proteins. The CYC428 and CYC408 complex datasets were obtained from the CYC2008 set from Wodak Lab [44, 45]. The four protein complexes were combined into a new complex for this study, consisting of 745 protein complexes.

### 4.2 Evaluation methods

Typically, several statistical measures, such as sensitivity (SN), specificity (SP), positive predictive value (PPV), negative predictive value (NPV), *F* ‐measure (*F*) and accuracy (ACC), are used to effectively determine the identification of essential proteins using different methods [10, 17]. These measures were used to assess the effectiveness of the proposed method, DENCI. The four variables are defined as follows:

- True positive (TP): essential proteins correctly selected as essential.
- False positive (FP): non‐essential proteins incorrectly selected as essential.
- True negative (TN): non‐essential proteins correctly selected as non‐essential.
- False negative (FN): essential proteins incorrectly selected as non‐essential.

Next, six statistical measures are introduced as mentioned above.

*Sensitivity* : the ratio of proteins accurately selected as essential to the total number of essential proteins

(12) $$\text{SN}=\frac{\text{TP}}{\text{TP}+\text{FN}}$$

*Specificity* : the ratio of non‐essential proteins accurately selected as non‐essential to the total number of non‐essential proteins

(13) $$\text{SP}=\frac{\text{TN}}{\text{TN}+\text{FP}}$$

*Positive predictive value* : the ratio of proteins accurately selected as essential

(14) $$\text{PPV}=\frac{\text{TP}}{\text{TP}+\text{FP}}$$

*Negative predictive value* : the ratio of proteins accurately selected as non‐essential

(15) $$\text{NPV}=\frac{\text{TN}}{\text{TN}+\text{FN}}$$

*F‐measure* : the harmonic mean of SN and PPV is referred to as *F* ‐measure

(16) $$F=\frac{2\times \text{SN}\times \text{PPV}}{\text{SN}+\text{PPV}}$$

*Accuracy* : the ratios of proteins correctly regarded as essential and non‐essential in all of the results

(17) $$\text{ACC}=\frac{\text{TP}+\text{TN}}{P+N}$$

where *P* denotes the number of essential and *N* shows the number of non‐essential proteins.

### 4.3 Comparison of the results of the proposed method with other prediction measures

**Fig. 2:**
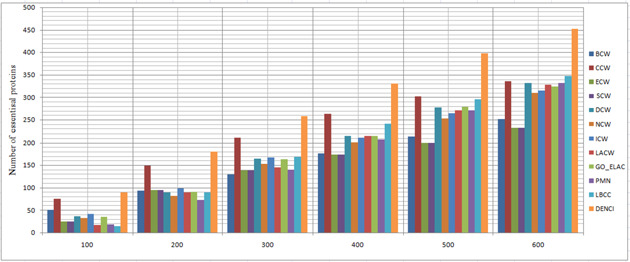
Number of true essential proteins predicted by DENCI and the other 11 previously proposed methods for the YHQ‐W network

**Fig. 3:**
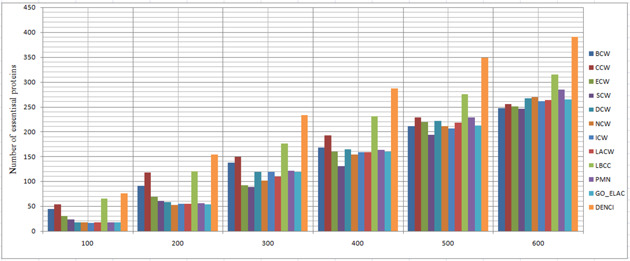
Number of true essential proteins predicted by DENCI and the other 11 previously proposed methods for the YMBD‐W network

**Fig. 4:**
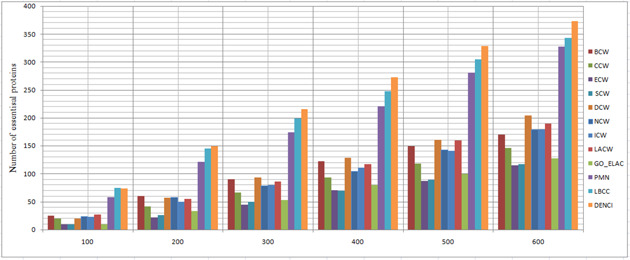
Number of true essential proteins predicted by DENCI and the other 11 previously proposed methods for the YMIPS‐W network

**Fig. 5:**
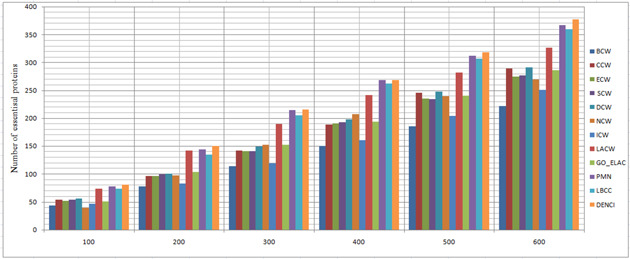
Number of true essential proteins predicted by DENCI and the other 11 previously proposed methods for the YDIP‐W network

In this section, the results of the proposed method are compared with those of the following three categories of prediction methods, using the four datasets described in the experimental data section. Using the combined classification measures of the proposed method, we obtained a value for each protein; this value shows the importance of each protein (vertex) compared to other proteins. The higher the importance of each protein, the higher the probability that the protein will be essential. Therefore, based on the calculated value from (11) for each protein, they were sorted downwards to predict the top 100 up to the top 600. Moreover, 20% of the dataset was selected as essential proteins. Next, we compared the top 100 to the top 600 essential protein with the essential proteins of the benchmark, to see how many of them were rightly identified. We measured the precision of identifying the essential proteins using statistical measures. The prediction results of the 12 methods for the four different networks are shown in Figs. 2, 3, 4–5.

Regarding the YHQ‐W dataset in Fig. 2, it is shown that except for DENCI, the highest number of essential proteins, 75(CCW), 149(CCW), 212(CCW), 263(CCW), 303(CCW) and 348 (LBCC), were identified on six levels ranging from the top 100 to the top 600. In comparison, DENCI had 90,180,258, 331, 399 and 452 correct essential proteins with precision prediction of at least 20, 20, 21, 25, 31 and 29% on six levels ranging from the top 100 to the top 600. Values of *a*, *b*, *c* and *d* in DENCI were equal to 0.143, 0.184, 0.358 and 0.313, respectively.

For the YMBD‐W dataset, Fig. 3 shows that the number of essential proteins predicted correctly by DENCI was 76, 154, 233, 287, 348 and 391, respectively, which is the best performance of all the methods. After the DENCI method, the most recent LBCC method has the second performance with 65, 120, 176, 231, 275 and 315 essential proteins correctly identified on six levels ranging from the top 100 to the top 600. DENCI performs better than LBCC, and the prediction precision increased by more than 28, 16, 32, 24, 26 and 24% on the six levels from the top 100 to the top 600. Values of *a*, *b*, *c* and *d* in DENCI were equal to 0.221, 0.338, 0.270 and 0.189, respectively. For the YMIPS‐W dataset, Fig. 4 shows that LBCC produced the best results on the top 100 level, while DENCI performed better on the five levels from the top 200 to the top 600. On all six levels, the number of essential proteins identified correctly by LBCC was 75, 145, 199, 248, 305 and 343. In comparison, the number of essential proteins identified correctly by DENCI was 74, 149, 216, 273, 328 and 373, respectively. In the top 100, the prediction results of DENCI were very similar to those of LBCC (<2% difference), and on the five levels from the top 200 to the top 600, the prediction precision increased by more than 2, 8, 10, 7 and 8%. Values of *a*, *b*, *c* and *d* in DENCI were equal to 0.239, 0.314, 0.298 and 0.148, respectively. For the YDIP‐W dataset, Fig. 5 shows that the number of essential proteins predicted correctly by DENCI was 81, 150, 216, 268, 319 and 377, respectively, which indicates the best performance. After the DENCI method, the PMN method had the second best performance with 78, 144, 214, 268, 313 and 367 essential proteins identified on six levels from the top 100 to the top 600. DENCI performs better than PMN, and the prediction precision increased by more than 4, 3, 1, 1 and 2% on the top 100, 200, 300, 500 and 600 levels. The precision of the two methods was only equal on the top 400 level. Values of *a*, *b*, *c* and *d* in DENCI were equal to 0.186, 0.275, 0.363 and 0.174, respectively. Therefore, our experiments show that DENCI can identify more proteins than other methods in all cases except for one case where it identifies the same number of proteins, and one case where it identifies 2% less. The reason for the success of the results is that the method simultaneously considers local and global centralities, as well as biological characteristics. The results of other methods (Figs. 2 and 5) show that local or global centrality methods perform better for dense components and methods using biological features perform better for sparse components with low connectivity. The proposed method has all of the previously mentioned benefits.

### 4.4 Validation by six statistical methods and the precision‐recall curve

In this section, DENCI is compared with 11 prediction measures in three categories listed by six statistical methods, as described in Section 4.2. The proteins were ranked in descending order based on the values of the 12 centrality measures. The top proteins (top 20%) were selected as essential and the rest were considered as non‐essential proteins. According to the results in Table 2, the values of the six statistical methods for DENCI were mostly higher than for the other methods, particularly for the three networks of YHQ‐W, YMBD‐W and YMIPS‐W, indicating that DENCI can correctly predict a higher number of essential proteins. Values of *a*, *b*, *c* and *d* in DENCI were equal to (0.143, 0.184, 0.358, 0.313), (0.221, 0.338, 0.270, 0.189), (0.239, 0.314, 0.298, 0.148) for the YHQ‐W, YMBD‐W and YMIPS‐W dataset, respectively.

**Table 2:** Comparative analysis of DENCI and the other 11 previously proposed methods in terms of SN, SP, PPV, NPV, *F* ‐measure and ACC with four different datasets

Dataset	Methods	SN	SP	PPV	NPV	*F* ‐measure	ACC
YMBD−W	BCW	0.256	0.881	0.431	0.771	0.321	0.718
	CCW	0.275	0.888	0.464	0.777	0.346	0.729
	ECW	0.257	0.881	0.431	0.773	0.322	0.719
	SCW	0.212	0.865	0.354	0.759	0.265	0.696
	DCW	0.248	0.878	0.417	0.769	0.311	0.714
	NCW	0.242	0.876	0.406	0.767	0.303	0.711
	ICW	0.237	0.874	0.397	0.766	0.296	0.709
	LACW	0.246	0.877	0.412	0.768	0.308	0.713
	LBCC	0.372	0.873	0.555	0.766	0.445	0.724
	PMN	0.252	0.879	0.424	0.770	0.316	0.717
	GO_ELAC	0.243	0.876	0.408	0.768	0.305	0.712
	DENCI	**0.425**	**0.941**	**0.717**	**0.822**	**0.533**	**0.806**
YHQ−W	BCW	0.295	0.864	0.404	0.797	0.341	0.729
	CCW	0.371	0.888	0.509	0.819	0.429	0.765
	ECW	0.273	0.857	0.370	0.793	0.315	0.720
	SCW	0.273	0.857	0.370	0.793	0.315	0.720
	DCW	0.383	0.892	0.524	0.822	0.443	0.771
	NCW	0.375	0.889	0.514	0.820	0.434	0.767
	ICW	0.362	0.885	0.495	0.817	0.418	0.761
	LACW	0.384	0.892	0.525	0.822	0.443	0.771
	LBCC	0.449	0.876	0.524	0.839	0.483	0.776
	PMN	0.388	0.894	0.533	0.824	0.449	0.773
	GO_ELAC	0.375	0.889	0.515	0.820	0.434	0.767
	DENCI	**0.475**	**0.921**	**0.652**	**0.849**	**0.549**	**0.815**
YMIPS−W	BCW	0.223	0.808	0.289	0.748	0.252	0.656
	CCW	0.200	0.800	0.263	0.738	0.227	0.643
	ECW	0.156	0.784	0.205	0.723	0.177	0.619
	SCW	0.161	0.786	0.212	0.725	0.183	0.622
	DCW	0.264	0.822	0.345	0.759	0.299	0.676
	NCW	0.250	0.817	0.324	0.757	0.282	0.670
	ICW	0.217	0.806	0.284	0.744	0.246	0.652
	LACW	0.238	0.813	0.309	0.752	0.269	0.664
	LBCC	0.430	0.866	0.481	0.841	0.454	0.769
	PMN	0.387	0.889	0.505	0.832	0.316	0.717
	GO_ELAC	0.243	0.876	0.408	0.768	0.305	0.712
	DENCI	**0.437**	**0.903**	**0.567**	**0.847**	**0.494**	**0.799**
YDIP−W	BCW	0.267	0.838	0.354	0.776	0.305	0.741
	CCW	0.343	0.863	0.456	0.798	0.392	0.781
	ECW	0.334	0.860	0.444	0.795	0.382	0.776
	SCW	0.337	0.862	0.447	0.796	0.384	0.777
	DCW	0.348	0.865	0.462	0.799	0.398	0.783
	NCW	0.335	0.860	0.442	0.797	0.381	0.777
	ICW	0.306	0.851	0.406	0.787	0.349	0.761
	LACW	0.367	0.871	0.484	0.806	0.418	0.794
	LBCC	0.410	0.873	0.512	0.811	0.454	0.776
	PMN	0.394	0.880	0.523	0.813	0.449	0.807
	GO_ELAC	0.347	0.865	0.460	0.799	0.397	0.782
	DENCI	**0.412**	**0.886**	**0.547**	**0.819**	**0.470**	**0.817**

The Precision‐Recall curve is a statistical method that assesses the stability of the 12 prediction measures. This curve is drawn as follows:

(18) $$\text{Precision}\left(n\right)=\frac{\text{TP}\left(n\right)}{\text{TP}\left(n\right)+\text{FP}\left(n\right)}$$

(19) $$\text{Recall}\left(n\right)=\frac{\text{TP}\left(n\right)}{P}$$

where TP(*n*) shows the total number of essential proteins correctly detected as essential and FP(*n*) indicates the total number of non‐essential proteins inaccurately recognised as being essential among the *n* th top identified proteins. The total number of essential proteins under consideration is named *P*.

**Fig. 6:**
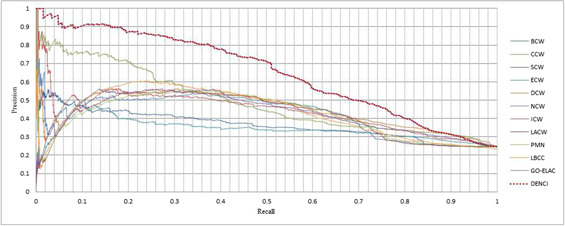
Precision and recall curves of DENCI and 11 other methods for the YHQ‐W network

The YHQ‐W network (Fig. 6) revealed that DENCI performed better than the other methods. The other networks revealed that DENCI performed better than the other methods.

In an analysis using six statistical methods and the precision‐recall curve, DENCI not only showed a better prediction precision compared to the other 11 methods, but it also showed a more stable efficiency, particularly in the three networks of YMBD‐W, YHQ‐W and YMIPS‐W.

### 4.5 Validation by the jackknife method

**Fig. 7:**
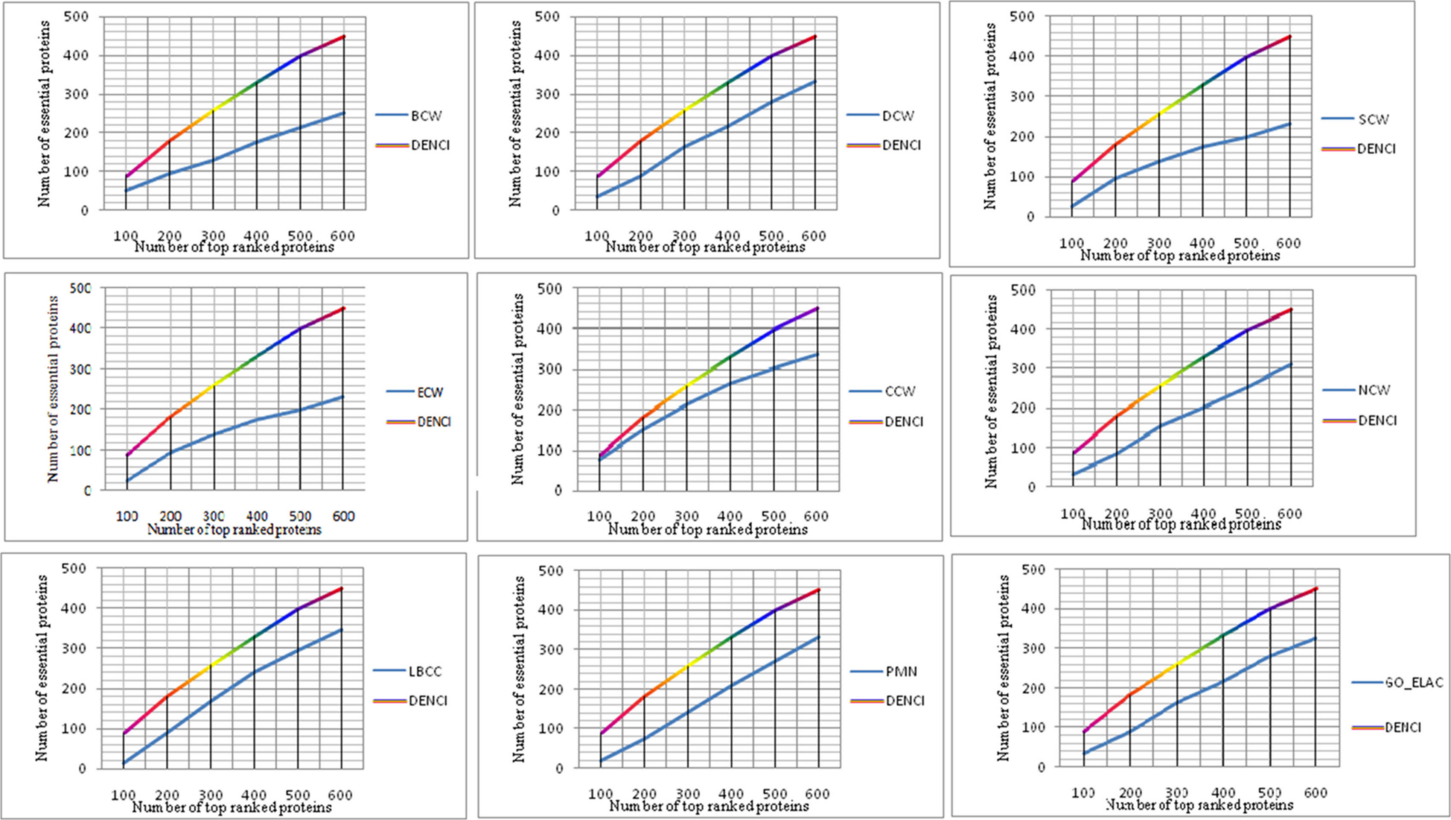
Jackknife curves of DENCI and 11 other methods for the YHQ‐W network

The jackknife method, developed by Hellman *et al.* [48], was used to evaluate all of the measures as a whole, including single‐centrality measures, combined centrality methods and methods based on measures of biological characteristics. First, the proteins were ranked in descending order for the values obtained through the 12 prediction methods. The jackknife curve was then drawn according to the cumulative number of essential proteins. As shown in Fig. 7, the *x* ‐axis shows that the proteins were ranked in descending order from left to right according to the values calculated by the specific prediction method. The *y* ‐axis represents the number of real essential proteins among the *n* th top proteins, where *n* is the numbers along the *x* ‐axis. As shown in Fig. 7, the curve arranged according to the DENCI method is significantly better than those arranged according to the other prediction measures in the three prediction method categories from the YHQ‐W datasets. Values of *a*, *b*, *c* and *d* in DENCI were equal to 0.143, 0.184, 0.358 and 0.313, respectively. Taking into consideration the curves, DENCI is an effective approach for the prediction of essential proteins.

### 4.6 Validation by the Receiver Operating Characteristic (ROC) curve

**Fig. 8:**
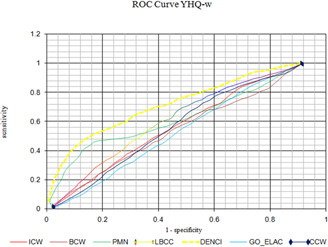
ROC curves of DENCI and the other methods for the YHQ‐W network

The ROC curve is a curve that shows SN against ‘1‐SP’. The curve is a suitable measure to estimate a method. The identification of essential proteins is a two‐class case of classification. This kind of classification is thought of as an imbalanced classification because the number of non‐essential proteins is twice or thrice that of the essential ones in PIN networks. Therefore, ROC can effectively estimate the performance of a method. As shown in Fig. 8, the ROC curve is higher for DENCI than for others in YHQ‐W. Values of *a*, *b*, *c* and *d* in DENCI were equal to 0.143, 0.184, 0.358 and 0.313, respectively. Taking into consideration the ROCs, our proposed method, DENCI, is an effective method for predicting essential proteins.

### 4.7 Analysis of the difference between DENCI and other measures

This section carefully discusses why DENCI functions well in the four datasets for the prediction of essential proteins. The differences between DENCI and the other prediction measures are examined using a few different proteins. If A∩B is considered as the set of proteins predicted by the two methods of *A* and *B*, then A−B and A∪B, will be the protein sets predicted by Method *A* but not predicted by Method *B*, and the set of proteins predicted by method *A* or *B*, respectively. The efficiency of DENCI was compared with that of the other 11 approaches in terms of the prediction of the top 100 proteins (Table 3). As can be seen, for the YHQ‐W dataset in column DENCI∩M, the overlap rate of proteins predicted by DENCI and the other 11 methods (DCW, BCW, ECW, CCW, LACW, NCW, SCW, ICW, LBCC, Go_ELAC and PMN) was <32%, whereas DENCI had no overlaps with ECW and SCW in the proteins predicted by these methods. The overlap rate between DENCI and LBCC was 16%. Values of *a*, *b*, *c* and *d* in DENCI were equal to 0.143, 0.184, 0.358 and 0.313, respectively. The fifth column shows the true essential proteins in the DENCI‐M dataset, while the sixth column represents those in the M‐DENCI dataset. The number of true essential proteins identified by DENCI was higher than that identified by the other prediction methods. Regarding YMBD‐W, the column DENCI∩M suggests that the overlap in the proteins predicted by DENCI and those predicted by the other 11 approaches is no more than 32%. The fifth and sixth columns indicate that DENCI identified a greater number of true essential proteins than did the other prediction methods, such as LBCC. Values of *a*, *b*, *c* and *d* in DENCI were equal to 0.221, 0.338, 0.270 and 0.189, respectively. Our proposed method is a multi‐information fusion measure (MCLUS, Importance, DWNN and LACW), and one of the features of our approach is that it also considers low‐connectivity essential proteins. The YMBD‐W dataset has a total of 61 components, consisting of 41 two‐protein components, ten three‐protein components, three four‐protein components, one five‐protein component, one eight‐protein component, one nine‐protein component, one ten‐protein component, one 31‐protein component and one 2053‐protein component. Therefore, there are many low‐connectivity proteins in this dataset. Our proposed method, which focuses on identifying low‐connectivity proteins using the two independent variables of MCLUS and Importance, identifies low‐connectivity essential proteins. Given that the YDIP‐W dataset has a total of 15 components, including 13 two‐protein components, one three‐protein component and one 4757‐protein component, the number of low‐connectivity essential proteins is therefore much less than in the YMBD‐W dataset. The proposed method and all of the methods discussed in Table 3 proteins in common.

**Table 3:** Analysis of the differences between DENCI and the other 11 previously proposed methods for predicting proteins, Tep, true essential protein; S1, DENCI‐M; S2, M‐DENCI

Dataset	Methods	$\left|\text{DENCI}\cap M\right|$	$\left|S1\right|$	Tep in S1	Tep in S2
YHQ−W	BCW	14	86	69	33
	CCW	32	68	56	35
	ECW	0	100	89	26
	SCW	0	100	89	26
	DCW	15	85	74	22
	NCW	9	91	80	24
	ICW	19	81	70	24
	LACW	11	89	78	41
	LBCC	16	84	73	50
	PMN	1	99	88	18
	GO_ELAC	15	85	74	21
YMBD−W	BCW	11	89	70	39
	CCW	32	68	56	35
	ECW	2	98	76	31
	SCW	2	98	76	31
	DCW	11	89	62	14
	NCW	11	89	78	41
	ICW	13	87	59	37
	LACW	5	95	12	42
	LBCC	18	82	62	14
	PMN	14	86	69	33
	GO_ELAC	17	83	66	16
YMIPS−W	BCW	15	88	58	11
	CCW	12	88	61	9
	ECW	0	100	73	10
	SCW	0	100	73	10
	DCW	11	89	73	10
	NCW	6	94	67	17
	ICW	12	88	61	12
	LACW	9	81	64	18
	LBCC	23	77	58	39
	PMN	38	62	35	21
	GO_ELAC	3	97	70	7
YDIP−W	BCW	14	86	69	30
	CCW	42	58	41	33
	ECW	20	80	63	33
	SCW	22	78	61	33
	DCW	24	76	59	33
	NCW	16	84	67	24
	ICW	14	86	69	33
	LACW	19	81	64	35
	LBCC	43	57	40	31
	PMN	68	32	15	11
	GO_ELAC	19	81	64	33

## 5 Conclusion

A number of methods has been recommended to identify essential proteins; however, most of them are based on the topological features of PPI networks. The majority of topology‐based methods only focus on the local and global properties and their SN to the network topology. Our research demonstrated an innovative method called DENCI, which is based on a combination of both neighbourhood and biological features measures for PPI networks. An analysis of DENCI showed that the neighbourhood measure was significant up to the third level. Then, DENCI and the other methods were implemented on four PPI networks, YMIPS‐W, YMBD‐W, YHQ‐W and YDIP‐W. Comparisons were made between DENCI, traditional methods, the most significant recent combined centrality method and the most important measures of biological features in terms of the number of true essential proteins that they were predicted. On the six levels from the top 100 to the top 600 proteins, DENCI performed better than traditional methods, as well as measures of biological features, especially for the YHQ‐W, YMBD‐W and YMIPS datasets. The prediction precision of the DENCI method was superior to the most efficient methods in each of the three categories. Experimental results in the four datasets, based on six statistical analyses, the Precision‐Recall, ROC curve and the jackknife method, indicate that DENCI was in most cases more efficient than the other recent prediction methods. In the future, additional information such as domain data and gene expression data should be integrated for the effective and accurate prediction of essential proteins.
